# The role of I-FABP as a biomarker of intestinal barrier dysfunction driven by gut microbiota changes in obesity

**DOI:** 10.1186/s12986-016-0089-7

**Published:** 2016-04-30

**Authors:** Eva Lau, Cláudia Marques, Diogo Pestana, Mariana Santoalha, Davide Carvalho, Paula Freitas, Conceição Calhau

**Affiliations:** Serviço de Endocrinologia, Diabetes e Metabolismo, Centro Hospitalar São João, Porto, Portugal; Instituto de Investigação e Inovação em Saúde, Porto, Portugal; Departamento de Bioquímica, Faculdade de Medicina, Universidade do Porto, Porto, Portugal; CINTESIS, Centro de Investigação em Tecnologias e Serviços de Saúde, Porto, Portugal; Departamento de Medicina, Faculdade de Medicina, Universidade do Porto, Porto, Portugal; Nutrição e Metabolismo, NOVA Medical School|Faculdade de Ciências Médicas, Universidade NOVA de Lisboa, Lisboa, Portugal

**Keywords:** Inflammation, Intestinal fatty acid binding protein, Intestinal permeability, Obesity, Gut microbiota

## Abstract

**Background:**

Intestinal fatty-acid binding protein (I-FABP) is expressed in epithelial cells of the mucosal layer of the small intestine tissue. When intestinal mucosal damage occurs, I-FABP is released into the circulation and its plasma concentration increases. In the context of obesity, the gut barrier integrity can be disrupted by dietary fat while intestinal permeability increases.

**Objective:**

To investigate whether intestinal fatty acid binding protein (I-FABP) is a suitable plasma marker of intestinal injury and inflammation in obesity.

**Methods:**

Twelve male Wistar rats were randomly divided into two groups of six animals each: standard (St) and high-fat (HF) diet fed groups for 12 weeks.

**Results:**

HF fed animals developed obesity, insulin resistance and seemed to present increased plasma levels of proinflammatory cytokines (MCP-1 and IL1β). The gut microbiota composition of these animals was also altered, with lower number of copies of Bacteroidetes, Prevotella spp. and Lactobacillus spp., in comparison with those from St diet group. Fecal lipopolysaccharide (LPS) concentrations tended to be increased in HF fed animals. Intestinal expression of TLR4 seemed to be also increased in HF fed animals suggesting that HF diet-induced dysbiosis may be behind the systemic inflammation observed. However, in contrast to other intestinal inflammatory diseases, plasma I-FABP levels were decreased in HF fed rats whereas I-FABP expression in jejunum tended to be increased.

**Conclusions:**

HF diet-induced obesity is characterized by dysbiosis, insulin resistance and systemic inflammation. In this context, plasmatic I-FABP should not be used as a marker of the intestinal barrier dysfunction and the low-grade chronic inflammatory status.

## Background

The new concepts on the pathophysiology of obesity and insulin resistance highlight the role of intestinal microbiota and intestinal barrier in the development of these disorders [1, 2]. Microbiota seems to mediate obesity and associated metabolic disturbances through several mechanisms including energy storage and metabolic inflammation [1, 3]. On the other hand, intestinal mucosa plays not only an important part in the absorption of vital nutrients, but also in anatomical/barrier and immune functions, preventing bacterial translocation. High-fat diet changes gut microbiota composition and increases intestinal permeability, by a mechanism associated with a reduced expression of epithelial tight junction proteins [4]. The altered intestinal barrier and the subsequent translocation of bacteria or bacterial products, namely lipopolysaccharide (LPS) are now recognized as key mediators of the low-grade inflammation state, which characterize metabolic disorders.

Intestinal fatty-acid binding protein (I-FABP) is an intracellular protein specifically and abundantly expressed in the epithelial cells of the mucosal layer of the small and large intestine tissue [5]. The location of I-FABP in the mature epithelium of villi facilitates its leakage into the circulation from enterocytes when intestinal mucosal damage occurs [5]. It has been shown that I-FABP is released into the circulation following small intestinal mucosal injury and its plasma concentration has been associated with small intestinal diseases - necrotizing enterocolitis and celiac disease [6–8]. Therefore, I-FABP has emerged as a possible non-invasive marker for evaluating gut wall integrity loss and inflammation. Defining new and early non-invasive markers of gut barrier dysfunction might be of great interest in order to manage a safe modulation of the intestinal microbiota before emergence of obesity and associated metabolic diseases.

The aim of this study was to investigate the role of I-FABP as a possible plasma marker of intestinal injury and inflammation and its relationship with microbiota dysbiosis in a high-fat diet-induced obesity Rat model.

## Methods

### Animals and housing

Twelve male Wistar rats were purchased from Charles River (Barcelona, Spain) and kept under controlled environmental conditions (22–24 °C and 12 h light/dark cycles), for at least 1 week before starting the experiments. Animals, 8 weeks of age, were randomly divided into two groups of six animals each: standard (St) and high-fat (HF) diet group. The diets were respectively “St” (Teklad 2014, Harlan Laboratories, Santiga, Spain) and “HF” with 45 % of energy from lipids and 17 % of energy from sucrose (D12451 Research Diets, New Brunswick, NJ, USA). Animals were subjected to different experimental conditions for a total of 12 weeks. The water and chow were supplied ad libitum. Food and beverage consumption and body weight were monitored weekly, to carefully characterize energy ingestion and weight gain.

At the end of the 12 weeks, food was removed 4–6 h before sacrifice and the animals were anesthetized with a mixture of ketamine (50 mg/kg) and medetomidine (1 mg/kg) and maintained with isoflurane. Meanwhile, using a Quantum/S bioelectrical impedance analyzer (RJL Systems, Akern SRL, Florence, Italy), the body composition of each rat was determined by bioelectrical impedance, according to the procedure already described in the literature [9]. Before perfusion of the vascular compartment with a saline solution (NaCl 0.9 %, w/v), blood was drawn from the left ventricle into tubes with or without heparin to obtain plasma and serum, respectively. Aliquots were frozen at −80 °C until further analysis. Colon and jejunum were dissected, pat dried and snap-frozen in liquid nitrogen. Fresh fecal samples were collected directly from the colon of all animals and snap-frozen in liquid nitrogen. Both tissues and fecal samples were stored at −80 °C until further analysis.

Animal handling and housing protocols followed European Union guidelines (Directive 2010/63/EU) for the use of experimental animals in scientific research. The protocol was approved by the Committee on the Ethics of Animal Experiments of the Faculty of Medicine of University of Porto.

### Oral glucose tolerance test (OGTT)

After 7 weeks of treatment, rats were fasted over 5 h and a baseline blood draw from the saphenous vein was collected for plasma fasting glucose and insulin measurements. Animals were gavaged with a glucose solution of 2 g/kg body weight and blood droplets from the saphenous vein were collected to measure glycaemia thereafter at 30, 60, 90 and 120 min. Glucose levels were measured with Precision Xtra Plus test strips and an Optium Xceed device (Abbott Diabetes Care, Ltd., Maidenhead, UK). Plasma insulin levels were measured using a Rat/Mouse Insulin ELISA kit (Merck Milipore, Madrid, Spain). The homeostasis model assessment (HOMA) was used to calculate approximate insulin resistance using the formula: glucose (mg/dL) × insulin (ng/mL)/405.

### Blood and biochemical analysis

Biochemical evaluation of plasma was performed at the end of the study at São João Hospital Center Clinical Pathology Department. Routine biochemical parameters were measured using conventional methods with an Olympus AU5400® automated clinical chemistry analyzer (Beckman-Coulter, Izasa, Porto, Portugal).

Plasma content in leptin, adiponectin, monocyte chemoattractant protein-1 (MCP-1), I-FABP and glucagon-like peptide-2 (GLP-2) were determined using, respectively, Rat Leptin ELISA Kit (Merck, Milipore, Madrid, Spain), Human/Mouse/Rat Adiponectin Enzyme Immunoassay Kit (RayBiotech, Norcross, GA, USA), Rat MCP-1 ELISA Kit (RayBiotech, Norcross, GA, USA), Rat (FABP2) ELISA Kit (Shanghai Sunred Biological Technology Co., Ltd, Shangai) and GLP-2 ELISA Kit (Merck, Milipore, Madrid, Spain). Serum interleukin-1 *beta* (IL-1β) was determined by Luminex assay using custom Miliplex Rat Kits (Merck Milipore, Madrid, Spain), according to the manufacturer’s protocols using the Luminex Xmap Multiplexing Technology platform.

### Tissue RNA isolation and qRT-PCR

Total RNA from jejunum and colon was isolated using NZYol reagent (NZYTech, Portugal) according to manufacturer’s instructions. RNA samples were treated with DNase I (RQ1 RNase-free DNase; Promega, Portugal). cDNA was synthesized from 1 μg of treated mRNA with NZY First-Strand cDNA Synthesis Kit (NZYTech, Portugal). Quantitative real-time polymerase chain reaction (qRT-PCR) was run on Lightcycler96 (Roche Applied Science, Indianapolis, ID, USA). Cycling conditions were as follows: denaturation (95 °C for 10 min), amplification and quantification (95 °C for 10 s, annealing temperature for 10 s and 72 °C for 10 s, with a single fluorescence measurement at the end of the 72 °C for a 10-s segment) repeated for 45 cycles and a final melting step with a temperature ramp from 60 to 97 °C. Rat-specific primer sequences (Sigma-Aldrich, St. Louis, MO, USA) used are described in Table 1. The Cq values obtained were transformed into relative quantification data using the formula 2^−(ΔCq)^.

**Table 1:** Primer sequences and real-time PCR conditions used for gene expression analysis by qRT-PCR

Gene name	Primer Sequence (5’-3’)	AT
I-FABP	ATGGAAAGGAGCTGATTGCT	59 °C
	TTGGCCTCCACTCCTTCATA	
TLR4	GATGCCTCTCTTGCATCTGG	60 °C
	TCATGAGGGATTTTGCTGAGA	
GAPDH	GGCATCGTGGAAGGGCTCATGAC	62 °C
	ATGCCAGTGAGCTTCCCGTTCAGC	

*AT* annealing temperature, *GAPDH* glyceraldehyde-3-phosphate dehydrogenase (housekeeping gene), *I-FABP* intestinal fatty acid binding protein, *TLR4* Toll-like receptor 4

### DNA extraction from stool

Genomic DNA was extracted and purified from stool samples using NZY Tissue gDNA Isolation Kit (NZYtech, Lisbon, Portugal) with some modifications. Briefly, feces (170–200 mg) were homogenized in TE buffer (10 mM Tris/HCl; 1 mM EDTA, pH 8.0) and centrifuged at 4000 x *g* for 15 min. The supernatant was discarded and the pellet was resuspended in 350 μL of buffer NT1. After an incubation step at 95 °C for 10 min, samples were centrifuged at 11000 x *g* for 1 min. Then, 25 μL of proteinase K were added to 200 μL of the supernatant for incubation at 70 °C for 10 min. The remaining steps followed manufacturer’s instructions. DNA purity and quantification were assessed with a NanoDrop spectrophotometer (Thermo Scientific, Wilmington, DE, USA).

### Microbial analysis of Rat stool by qRT-PCR

qRT-PCR was performed in sealed 96-well microplates using a LightCycler FastStart DNA Master SYBR Green kit and a LightCycler96 instrument (Roche Applied Science, Indianapolis, ID, USA). Primer sequences (Sigma-Aldrich, St. Louis, MO, USA) used to target the 16S rRNA gene of the bacteria and the conditions for PCR amplification reactions were previously described in Marques C et al. [10]. To verify the specificity of the amplicon, a melting curve analysis was performed via monitoring SYBR Green fluorescence in the temperature ramp from 60 to 97 °C. Data were processed and analyzed using the LightCycler software (Roche Applied Science, Indianapolis, ID, USA). Standard curves were constructed using serial tenfold dilutions of bacterial genomic DNA, according to the following webpage http://cels.uri.edu/gsc/cndna.html. Bacterial genomic DNA used as a standard was obtained from DSMZ (Braunschweig, Germany). Genome size and the copy number of the 16S rRNA gene for each bacterial strain used as a standard was obtained from NCBI Genome database (www.ncbi.nlm.nih.gov). Data are presented as the mean values of duplicate PCR analysis.

### Fecal LPS quantification

Quantification of LPS was performed using the Chromo-Limulus Amebocyte Lysate (Chromo-LAL) reagent (Associates of Cape Cod, Inc., Falmouth, MA, USA). Briefly, 1 mL of sterile saline solution (NaCl 0.9 %) was added to 100 mg feces, vortexed and centrifuged (10 min, 10000 *g*, 4 °C) twice. Total supernatant (fecal water) was filtered with 0.45 μm filter and then with 0.22 μm filter. Fecal water and Chromo-LAL (1:1) were incubated at 37 °C for 20 min and absorbance was read every 10 s at 405 nm.

### Statistical analysis

Values are expressed as the arithmetic mean ± standard error of the mean (SEM). Given the small sample size, a non-parametric test (Mann–Whitney test) was used to analyze the differences between St and HF groups. Correlation between variables was established using two-tailed Pearson’s correlation test. The differences were considered statistically significant when *P* <0.05. All statistical analyses were performed using GraphPad Prism 6 statistical software (GraphPad Software Inc., La Jolla, CA, USA).

## Results

### Energy and metabolic parameters

Energy intake was increased in the HF diet fed group when compared with St diet fed group (72.60 ± 0.15 vs. 57.9 ± 0.47 kcal/day, *P* <0.05). Consequently, HF diet fed animals gained more weight during the 12 weeks of the study (205.5 ± 37.8 vs. 134.7 ± 22.5 g, *P* <0.05) (Table 2). To assess whether these differences in weight gain were related to alterations in adiposity, we decided to evaluate the body composition of the animals of both groups. Our results showed that rats under HF diet had more body fat mass comparing to rats under St diet (206.3 ± 21.03 vs. 166.8 ± 21.26, *P* <0.05).

**Table 2:** Energy ingestion, body composition and metabolic parameters of Wistar rats fed either with standard (St) or high-fat (HF) diet during 12 weeks

	St	HF	*P* value
Energy ingested (Kcal/day)	57.9 ± 0.5	72.60 ± 0.1	<0.05
Weight gain (g)	134.7 ± 9.2	205.5 ± 15.4	<0.05
Fat mass (g)	166.8 ± 8.7	206.3 ± 8.6	<0.05
Total cholesterol (mg/dL)	64.3 ± 3.1	69.8 ± 6.6	0.78
Triglycerides (mg/dL)	79.3 ± 11.5	79.2 ± 8.3	0.83
Leptin (ng/mL)	6.2 ± 1.2	19.1 ± 2.6	<0.05
GLP-2	7.0 ± 0.4	9.8 ± 0.8	<0.05

Values are presented as mean ± SEM (*n* = 6 rats per group). *GLP-2* glucagon-like peptide-2

**Fig. 1:**
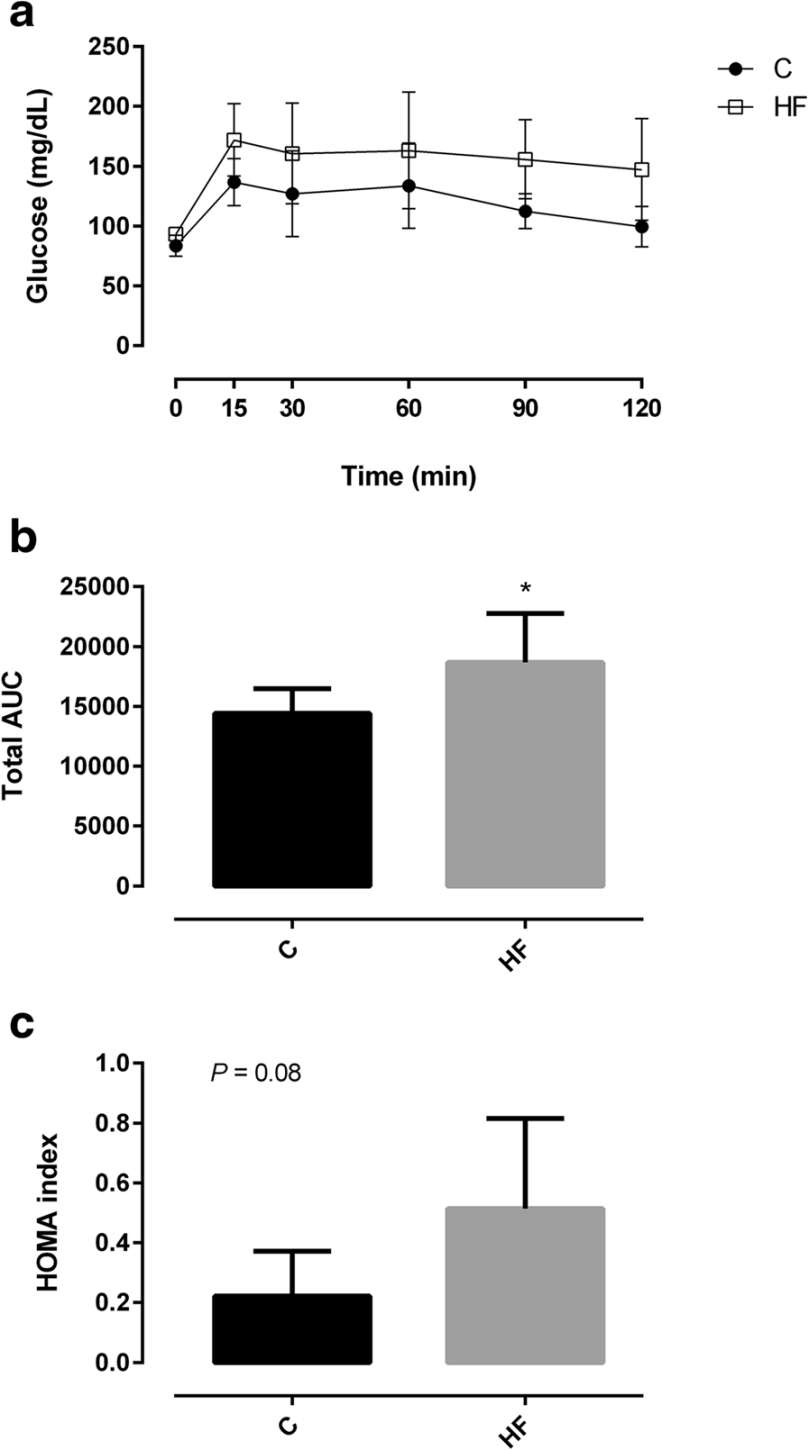
Glycaemic response during oral glucose tolerance test (**a**), total area under the curve (AUC) (**b**) and homeostasis model assessment (HOMA) (**c**) of Wistar rats after 7 weeks of feeding either with standard (St) or high-fat (HF) diet. HOMA was calculated using the formula: fasting glucose (mg/dL) × fasting insulin (ng/mL)/405. Data are presented as mean ± SEM (*n* = 6 rats per group). * *P* < 0.05 vs respective St diet group

To assess glycemic response, we performed an oral glucose tolerance test (OGTT). The total area under the curve (AUC) of the glycemic response was increased by HF diet feeding (Fig. 1a, b). In addition, HF diet fed rats had almost two fold less insulin sensitivity, as determined by homeostasis model assessment (HOMA) of insulin resistance (Fig. 1c).

We also examined plasma concentrations of leptin, which has postulated roles in obesity and insulin action (Table 2). HF diet fed group presented significantly higher leptin levels (19.1 ± 2.6 vs. 6.2 ± 1.2, *P* <0.05) than St diet fed group.

Total cholesterol and triglycerides did not differ between groups (Table 2).

### Gut microbiota, LPS and inflammatory status

To study whether HF diet could induce modifications within the intestinal microbiota, we quantified some of the main bacterial groups presented in fecal samples. Analysis of the bacterial 16S rDNA revealed that, at the phylum level, animals from the HF diet group were characterized for having lower Bacteroidetes and higher Firmicutes to Bacteroidetes ratio (Table 3). Firmicutes/Bacteroidetes ratio was positively correlated with weight gain (*r* = 0.829, *P* <0.05) and AUC (*r* = −0.723, *P* <0.05) while Bacteroidetes were negatively correlated with weight gain (*r* = −0.800, *P* <0.05) and AUC (*r* = −0.716 *P* <0.05).

**Table 3:** Quantification of gut microbiota phyla, genera and species in different experimental groups

	St	HF	*P* value
Firmicutes/Bacteroidetes	1.03 ± 0.01	1.20 ± 0.03	<0.05
Firmicutes	6.61 ± 0.08	6.50 ± 0.09	0.35
Bacteroidetes	6.43 ± 0.10	5.45 ± 0.15	<0.05
Bacteroides spp.	4.36 ± 0.23	4.15 ± 0.50	0.18
Prevotella spp.	3.59 ± 0.29	2.21 ± 0.13	<0.05
Lactobacillus spp.	4.86 ± 0.27	3.77 ± 0.17	<0.05
*Clostridium leptum*	5.53 ± 0.04	5.43 ± 0.12	0.65
Bifidobacterium spp.	2.00 ± 0.24	2.12 ± 0.22	0.75

Values are presented as mean ± SEM and expressed as log_10_ 16S rRNA gene copies/20 ng of DNA (*n* = 6 rats per group). *HF* high-fat diet group, *St* standard diet group

**Fig. 2:**
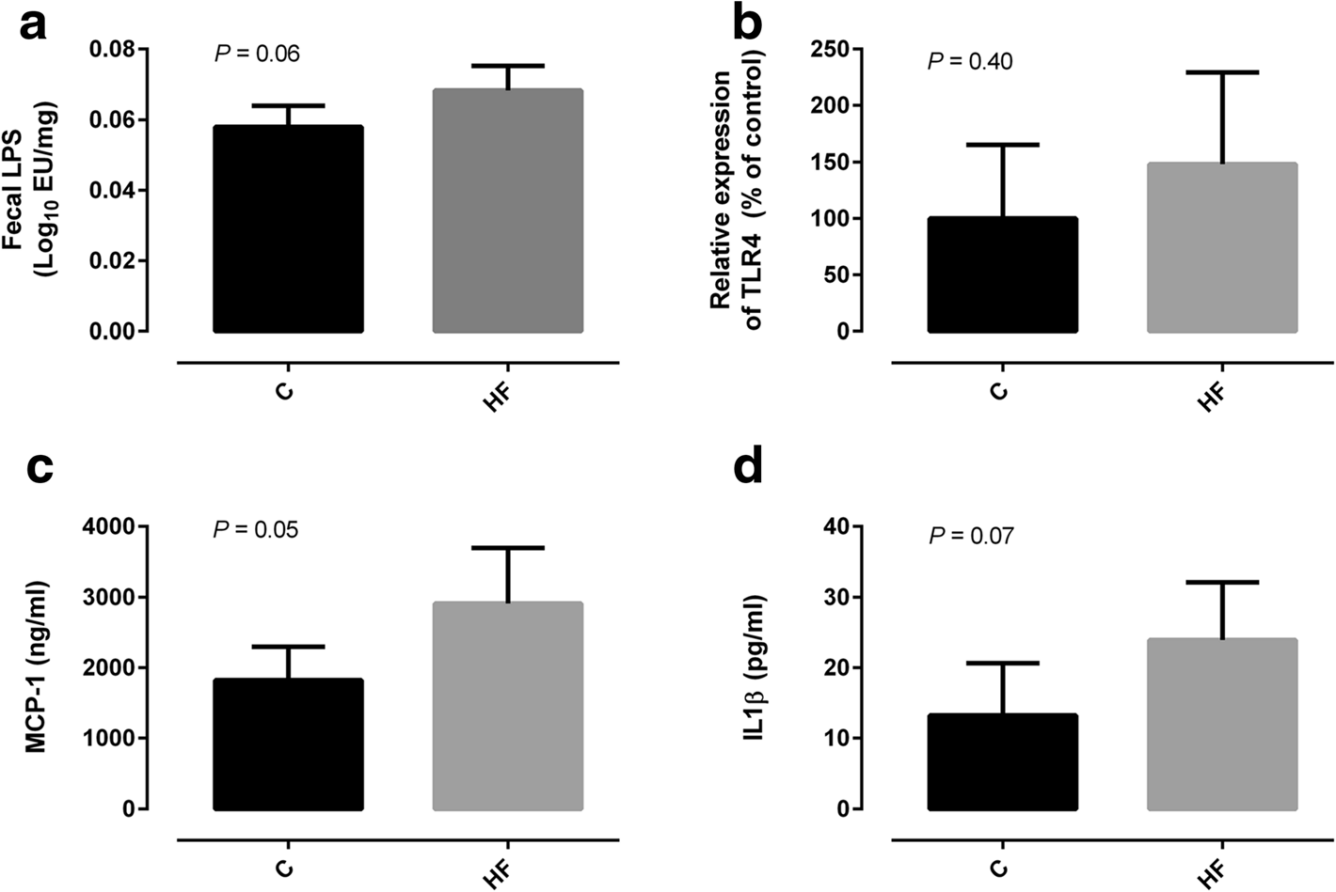
Fecal lipopolysaccharide (LPS) (**a**), intestinal expression of toll-like receptor 4 (TLR4) (**b**), plasma monocyte chemoattractant protein-1 (MCP-1) (**c**) and serum interleukin-1 *beta* (IL-1β) (**d**) of Wistar rats fed either with standard (St) or high-fat (HF) diet during 12 weeks. Data are presented as mean ± SEM (*n* = 3–6 rats per group). * *P* < 0.05 vs respective St diet group

HF diet feeding also resulted in a decrease in the number of copies of Prevotella spp. and Lactobacillus spp. (Table 3). We further determined whether fecal LPS levels could be altered as a result of the changes in the gut microbiota. Our data showed that fecal LPS levels seemed to be more elevated in HF diet fed animals (Fig. 2a). In addition, LPS levels were positively correlated with Firmicutes/Bacteroidetes ratio (*r* = 0.787, *P* <0.05) and negatively correlated with Bacteroidetes (*r* = −0.670, *P* <0.05).

Afterwards, we evaluated the colonic expression of toll-like receptor 4 (TLR4) which is capable to recognize LPS. In agreement with our LPS findings, TLR4 expression tended to be increased in the colon of animals fed with HF diet (Fig. 2b). Next, we investigated whether HF diet and microbiota changes were associated with systemic inflammation. In accordance, the chemokines MCP-1 and Il-1β appeared to be more elevated in the plasma of HF diet fed rats (Fig. 2c, d). LPS was positively correlated with MCP-1 (*r* = 0.726, *P* <0.05).

### I-FABP and GLP-2 in high fat-diet induced obesity

**Fig. 3:**
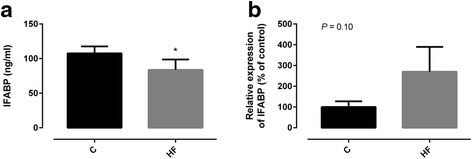
Levels of plasma (**a**) and intestinal expression of intestinal fatty-acid binding protein (I-FABP) (**b**) of Wistar rats fed either with standard (St) or high-fat (HF) diet during 12 weeks. Data are presented as mean ± SEM (*n* = 3–6 rats per group). * *P* < 0.05 vs respective St diet group

To understand the relationship between I-FABP, HF diet feeding and systemic inflammation we quantified plasma I-FABP levels and its intestinal expression in both groups of rats. Surprisingly, plasma I-FABP levels were decreased after HF diet feeding (Fig. 3a). On the other hand, I-FABP relative expression in jejunum tended to be higher in HF diet fed rats (Fig. 3b) which could be considered an adaptive response to the increased dietary fat content of the diet. To determine intestinotrophic status of the animals we quantified GLP-2 plasma levels. GLP-2 plasma levels were significantly increased after HF diet feeding (Table 2). GLP-2 and intestinal expression of I-FABP seemed positively correlated with energy ingested (*r* = 0.719, *P* <0.05 and *r* = 0.770, *P* = 0.07, respectively).

**Fig. 4:**
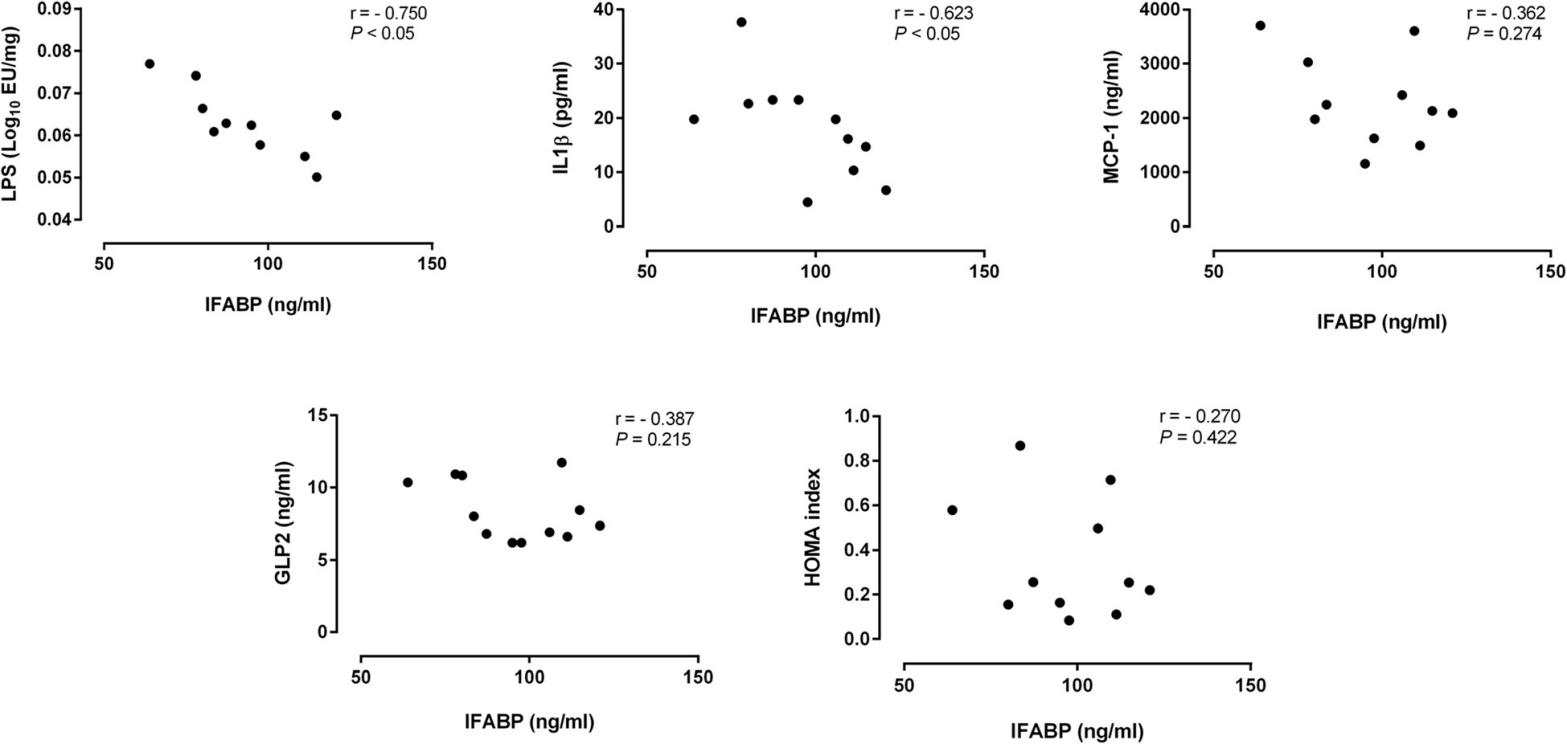
Correlations between plasma levels of intestinal fatty-acid binding protein (I-FABP) and host metabolic and inflammatory parameters. Data of all experimental groups were gathered and analyzed using two-tailed Pearson’s correlation test

To determine whether I-FABP plasma levels could be used as a marker of the metabolic alterations and inflammatory status associated with obesity, we evaluated the correlation between plasma I-FABP and host metabolic and inflammatory parameters (Fig. 4). Plasma I-FABP levels were negatively correlated with fecal LPS (*r* = −0.806, *P* <0.05) and IL-1B (*r* = −0.623, *P* <0.05).

## Discussion

Several studies have provided compelling evidence suggesting an association between gut microbiome dysbiosis, obesity and low-grade inflammatory state [11].

In consonance with previous reports, we found that the animals fed with HF diet had lower Bacteroidetes and higher Firmicutes to Bacteroidetes ratio [10]. This dysbiosis pattern might lead to an increased capacity of harvesting energy from food [12, 13]. HF diet also induced obesity and may have triggered intestinal inflammation since fecal levels of LPS and TLR4 expression tended to be increased in the animals fed with this diet. TLR4 is the LPS primary receptor that mediates its proinflammatory effects [14]. As a result, HF fed animals seemed to present higher plasma levels of proinflammatory cytokines and developed insulin resistance. As supported by other authors, it appears to be a causative role for the gut bacteria-induced proinflammatory state to the development of weight gain and insulin resistance in rats under HF diet [15, 16]. In this context, the gut barrier has an important role in the prevention of LPS leakage from the intestinal lumen to the portal blood. However, in animal models of diet-induced obesity, intestinal barrier function seems to be compromised [17].

In different intestinal diseases, I-FABP has emerged as a potential biomarker of intestinal barrier dysfunction [18, 19]. Basal I-FABP plasma levels may reflect the physiological turnover rate of enterocytes, whereas elevated levels might indicate intestinal epithelial cell damage [20]. Nevertheless, in the case of obesity-associated metabolic diseases, the existent data about I-FABP and intestinal barrier dysfunction is limited.

Verdam et al. have reported that chronically elevated glucose levels in obese individuals were associated with increased enterocyte loss, assumed by the increase on I-FABP levels [21]. It was therefore speculated that the increased enterocyte loss observed in subjects with chronic hyperglycemia might had contributed to the impaired intestinal barrier function, thereby promoting endotoxin-induced low-grade inflammation. However, increased I-FABP levels could also be a result of an increased production of I-FABP by enterocytes rather than enterocyte loss.

Interestingly, in our study we found that the relative expression of I-FABP tended to be increased in HF diet fed rats. As intestinal absorption capacity can be adapted to the dietary fat content, we hypothesize that HF diet may had up-regulated several genes known to play an important role in long-chain fatty acids uptake such as I-FABP [22]. The animals fed with HF diet also showed increased GLP-2 levels. GLP-2 is a 33 amino acid peptide associated with intestinal growth and adaptation in a variety of pathological conditions [23]. As suggested by other authors, GLP2/GLP2R system may be increased after HF diet to further promote fat absorption in the intestine [23]. On the other hand, the inflammatory state induced by microbiota changes after HF diet feeding might have increased GLP-2 production in order to improve the mucosal barrier integrity and, therefore, blunt the inflammatory stress [2].

## Conclusions

To our best knowledge, this is the first study demonstrating that, inversely to what happens in other intestinal inflammatory diseases, plasma I-FABP does not positively correlates with the inflammatory status presented in obesity. Instead, I-FABP is decreased in plasma but probably increased in jejunum in order to face dietary fat content. The search for noble biomarkers has to continue since it is extremely important to anticipate the progression of obesity-associated metabolic diseases and, thus, allowing the prevention or monitoring of the therapeutic strategies use.

## Abbreviations

AUC: area under the curve
GLP-2: glucagon-like peptide-2
HF: high-fat
HOMA: homeostasis model assessment
I-FABP: intestinal fatty-acid binding protein
IL-1β: interleukin-1 beta
LPS: lipopolysaccharide
MCP-1: monocyte chemoattractant protein-1
OGTT: oral glucose tolerance test
qRT-PCR: quantitative real-time polymerase chain reaction
St: standard
TLR4: toll-like receptor 4
